# Understanding the Role of Community Ecology and Pathogen Dynamics in Infectious Diseases in Animals

**DOI:** 10.3390/ani13030536

**Published:** 2023-02-02

**Authors:** João R. Mesquita

**Affiliations:** 1 ICBAS—School of Medicine and Biomedical Sciences, Porto University, 4050-313 Porto, Portugal; jrmesquita@icbas.up.pt; 2 Epidemiology Research Unit (EPIUnit), Instituto de Saúde Pública da Universidade do Porto, 4050-600 Porto, Portugal; 3 Laboratory for Integrative and Translational Research in Population Health (ITR), 4050-600 Porto, Portugal

Since the beginning of ages, pathogens have circulated worldwide, with many causing significant morbidity and mortality in humans [1]. Much of the diseases that these agents cause are known to be transmitted between animal species and between animals and humans, of which smallpox is a good example, having evolved from pathogens circulating in wildlife. In fact, nearly 70% of all emerging human infectious diseases are considered to have wildlife hosts or vectors [2].

A frequent cause playing a role in infectious diseases emergence is the involvement of multiple host, vector, or infectious agent in complex ecological communities, hampering control and mitigation efforts that target particular hosts for management [3]. Despite the multihost nature that infections may have, one can expect complex interactions among agents co-infecting the same host that can halter the pathology, transmission and virulence, with examples such as HIV that has favored drug-resistant forms of tuberculosis reemergence; and helminthiasis such as hookworm that can aggravate the outcome of malaria [4,5].

Besides this, ecological changes can sometimes drive evolution and change the complex interplay between hosts-environment-pathogens, ultimately favoring transmission. A good example can be seen in the El Niño events in the 90’s that led to the emergence of human hantavirus cases in the US, through an ecological cascade: increased precipitation led to growth of vegetation, which sustained increased populations of rodents, facilitating hantavirus transmission between rodents and from rodents to humans.

In this sense, community ecology wraps up the complex interplay and multitude of factors that generate the emergence of infectious agents in animals by examining the interactions between pathogens and their host organisms within an ecological context. It provides a pathway to understand how the dissemination and impact of infectious diseases is affected by factors such as population density, biodiversity, and environmental conditions, and how these factors in turn affect the distribution and evolution of species. This field of study helps bridge the gap between the micro-scale processes of individuals and populations and the macro-scale patterns of species distribution and evolution.

Disease community ecology hence combines the disciplines of epidemiology and community ecology to address the multispecies nature of many contemporary disease threats. The approach focuses on identifying the factors that govern the structure and dynamics of communities composed of multiple hosts, vectors, and symbionts, isolating the drivers of heterogeneity, and understanding how processes and patterns link across multiple scales of biological organization. A broader knowledge of these factors will facilitate more effective management of emerging infections, as complete eradication may not be successful [6].

This issue plans to collect the most recent advances in Animal infectious diseases epidemiology. Original research articles and comprehensive reviews that cover the community ecology and pathogen dynamics, including molecular aspects, transmission, infection, and pathology are welcomed.

## Data Availability

Data sharing not applicable.
